# Enhancing the Antioxidant Ability of *Trametes versicolor* Polysaccharopeptides by an Enzymatic Hydrolysis Process

**DOI:** 10.3390/molecules21091215

**Published:** 2016-09-10

**Authors:** Mei-Hsin Jhan, Ching-Hua Yeh, Chia-Chun Tsai, Ching-Tian Kao, Chao-Kai Chang, Chang-Wei Hsieh

**Affiliations:** 1 Department of Medicinal Botanicals and Health Applications, Da-Yeh University, No.168, Xuefu Rd., Dacun Township, Chang-Hua 51591, Taiwan; mickey810514@gmail.com (M-H.J.); judyyeh@mail.dyu.edu.tw (C-H.Y.); jasmine312034@hotmail.com (C-C.T.); 2 Department of Pediatrics, Cheng Ching General Hospital, Taichung 400, Taiwan; monster0936@yahoo.com.tw; 3 Biotechnology Research Center, Da-Yeh University, 168 University Rd, Dacun, Chang-Hua 51591, Taiwan; kai0913077636@gmail.com

**Keywords:** polysaccharopeptides (PSPs), enzymatic hydrolysates, antioxidant activities

## Abstract

Polysaccharopeptides (PSPs) are among the main bioactive constituents of *Trametes versicolor (T. versicolor)*. The purpose of this research was to investigate the antioxidant activities of enzymatic hydrolysates obtained from *T.* *versicolor* polysaccharopeptides by 80 U/mL β-1,3-glucanase (PSPs-EH80). The half-inhibitory concentration (IC_50_) of PSPs-EH80 in metal chelating assay, ABTS and DPPH radical scavenging test results were 0.83 mg/mL, 0.14 mg/mL and 0.52 mg/mL, respectively, which were lower than that of PSPs-EH 20 U/mL. The molecular weights of the PSPs-EH80 hydrolysates were 300, 190, 140 and 50 kDa, respectively, and the hydrolysis of polysaccharides by β-1,3-glucanase did not change the original functional group. PSPs-EH80 reduced the reactive oxygen species (ROS) content at least twice that of treatment without PSPs-EH80. In addition, an oxidative damage test showed that PSPs-EH80 can improve HaCaT cell survival. According to our results, PSP demonstrates the potential of anti-oxidative damage; besides, enzyme hydrolysis can improve the ability of the PSP.

## 1. Introduction

*Trametes versicolor (T. versicolor)* is a basidiomycete that has been used as a traditional medicinal mushroom [1]. Polysaccharides from *T. versicolor* have been reported to exhibit excellent bioactivity [2]. The protein-bound polysaccharides, polysaccharopeptides (PSPs), have been reported to be nontoxic with prolonged use in the treatment of cancers by suppressing DNA/RNA synthesis and enhancing the immune function [3,4,5].

The biological activities of polysaccharides are related to molecular weight, structure and viscosity [6,7]. To date, research has proved that the molecular weight of polysaccharides has a great influence on their biological activities [8]. For instance, after acidic degradation, low molecular weight polysaccharides exhibit stronger antioxidant activities than high molecular weight polysaccharides [9,10]. Conceptually, the degradation of high molecular weight polysaccharides might improve their poor penetration capability on cell membranes, thereby enhancing their activities [11].

Several chemical and physical methods, including enzymatic hydrolysis, acid hydrolysis and oxidative degradation, can break polysaccharides into lower molecular weight units [12,13]. Acid hydrolysis requires a long reaction time; it changes the structure of sugar units and can destroy necessary bioactive groups. Enzymatic hydrolysis can specifically cleave the glycosidic bonds in a polysaccharide chain [10,14]. Glucanases can be divided into exo- and endo- depending on the different positions of the glucan bonds they hydrolyze [15]. β-1,3-Glucanase, an endoglucanase widespread in bacteria, yeast, fungi and plants, hydrolyzes 1,3 bonds, producing oligosaccharides of different molecular weights.

In this study, to specifically hydrolyze β-(1→3)-glycosidic linkages in PSPs, β-(1→3)-d-glucanase was used to obtain enzymatic hydrolysates from *T. versicolor* PSPs. The antioxidant activities and functional groups of different molecular weight enzymatic hydrolysates were analyzed. The use of enzymatic hydrolysates from *T. versicolor* PSPs to counter oxidative damage by human keratinocytes (HaCaT) was also discussed in relation to evaluating the possibility of its development for use in the skin care industry.

## 2. Results and Discussion

### 2.1. Metal Chelating Activity

**Figure 1:**
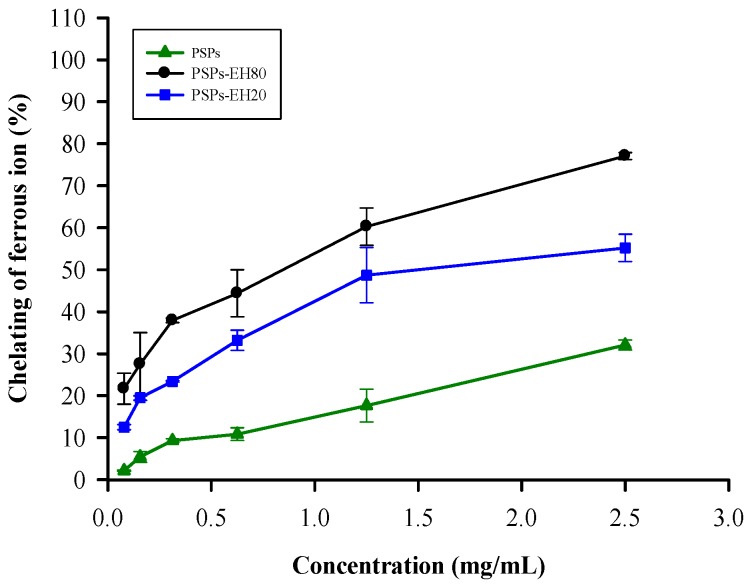
Antioxidant effects of PSPs and PSPs-EH on ferrous ion chelating ability. The ferrous ion chelating ability of PSPs and PSPs-EH was examined by reacting ferrozine with Fe^2+^; the color change was determined by measuring the absorbance at 562 nm. Error bars represent SD (*n* = 3), *p* < 0.05.

Ferrous ions (Fe^2+^) accelerate free radical accumulation in cells via the Fenton reaction (Fe^2+^ + H_2_O_2_ → Fe^3+^ + OH^−^ + •OH) [16]. Ferrozine can form complexes with Fe^2+^, disrupting the complex formation and resulting in the decreased red color of the complexes in the presence of other chelating agents [17]. We determined the antioxidant activities of PSPs, PSPs-EH20 and PSPs-EH80 using the Fe^2+^ chelating test. The ferrous ion chelating ability of PSPs-EH80 was better than that of PSPs-EH20 and PSPs, as shown in Figure 1. Relevant research has indicated that the *Enteromorpha prolifera* and *Ganoderma lucidum* polysaccharides of low molecular weight are more efficacious than those of higher molecular weight with respect to their Fe^2+^ chelating ability [10,18]. We indicate that the IC_50_ of the PSPs-EH80 hydrolyzed by enzyme is 0.83 mg/mL, which is 2–4 times better than *Enteromorpha prolifera* (IC_50_ is 2 mg/mL) and *Ganoderma lucidum* (IC_50_ is 4 mg/mL) [10,18].

### 2.2. ABTS Radical Scavenging Activity

**Figure 2:**
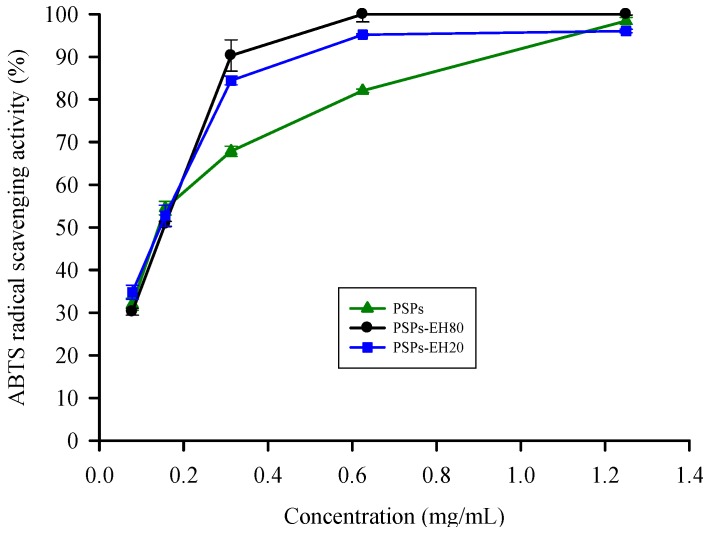
Antioxidant effects of PSPs and PSPs-EH on ABTS•^+ ^radical scavenging activity. The color change was determined by measuring the absorbance at 735 nm. Error bars represent SD (*n* = 3), *p* < 0.05.

The ABTS radical scavenging activity assay involves a reversible reduction-oxidation process. We determined the ABTS•^+^ scavenging ability of PSPs, PSPs-EH20 and PSPs-EH80 (the scavenging activity at 0.6 mg/mL is 70%, 90% and 100%, respectively) and found that the IC_50_ of PSPs-EH80 ABTS•^+^ scavenging ability was 0.14 mg/mL, which is lower than that of PSPs-EH20 (IC_50_ = 0.16 mg/mL) and PSPs (Figure 2). Furthermore, the PSPs-EH80 ABTS•^+^ scavenging ability has a better result than that of the floral mushroom and *Schisandra chinensis* polysaccharide [19,20]. This result suggests that PSPs-EH80 has a greater ability than PSPs-EH20 and PSPs to neutralize free radicals by accepting or donating an electron to eliminate the unpaired condition due to PSPs-EH80 having a low molecular weight [9]. Some natural polysaccharides are bound to protein or peptide residues, and the PSPs isolated from mushrooms were reported to have better radical scavenging activities [21,22]. Therefore, the radical scavenging activity of PSPs was better than that of polysaccharides. In this study, the ABTS•^+^ scavenging ability of PSPs-EH was even better than that of PSPs, which means that the hydrolysates are an effective natural antioxidant.

### 2.3. DPPH Radical Scavenging Activity

**Figure 3:**
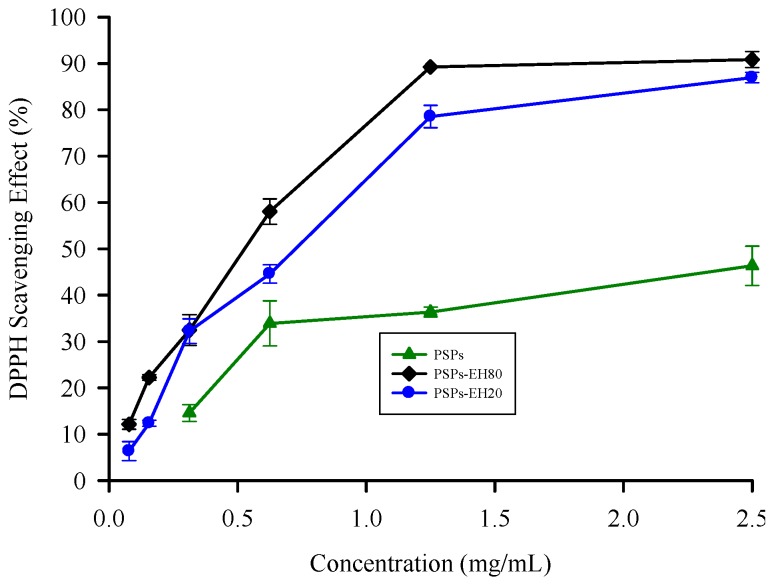
Antioxidant effects of PSPs and PSPs-EH on DPPH radical scavenging activity. The DPPH radical scavenging activity of PSPs and PSPs-EH was examined by reacting with DPPH radical, and the color change was determined by measuring the absorbance at 517 nm. Error bars represent SD (*n* = 3), *p* < 0.05.

Antioxidants inhibit oxidation chain reactions by providing hydrogen to DPPH radicals. The reaction can be expressed as follows: DPPH• + AH (antioxidant agent) → DPPH + HA•. The DPPH radical scavenging assay is often used to evaluate the hydrogen-providing ability of antioxidants [23]. The effects of PSPs and PSPs-EH on DPPH radical scavenging were determined as shown in Figure 3. The scavenging activity of PSPs, PSPs-EH20 and PSPs-EH80 is concentration-dependent. The enzymatic hydrolysates show the maximum scavenging (about 90% inhibition) at 1.2 mg/mL. The results revealed that enzymatic products of PSPs showed more effective DPPH radical activity than did the intact PSPs. Indeed, the DPPH radical scavenging activity of PSPs-EH80 was 2.5-fold higher than that of PSPs, with IC_50_ value of 0.52 mg/mL. The result could be explained by the fact that the enzymatic hydrolysates, PSPs-EH, could provide more hydrogen than the PSPs to form a stable DPPH-H molecular structure [9]. 

A previous study used enzymatic hydrolysates of polysaccharides to enhance the antioxidant ability [24]; however, using hydrolysis of specific bond of *T. versicolor* (mushroom polysaccharide) by β-1,3-glucanase to reduce the low molecular weight resulted in a 2.5-fold higher capacity. Furthermore, the lowest molecular weight fraction exhibited the highest DPPH radical and hydroxyl radical scavenging activities [25]. Therefore, we analyzed the molecular weight and the functional groups of PSP, as well as the antioxidant capacity of the best PSP-EH80 which has the strongest antioxidant ability, to determine the reason for the hydrolytic influencing the physiological activity.

### 2.4. Molecular Weight and FT-IR Spectroscopy

**Figure 4:**
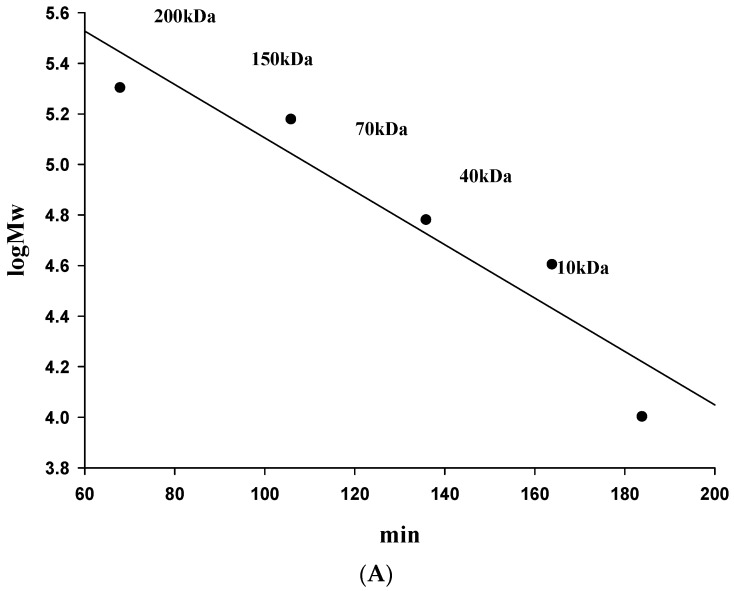

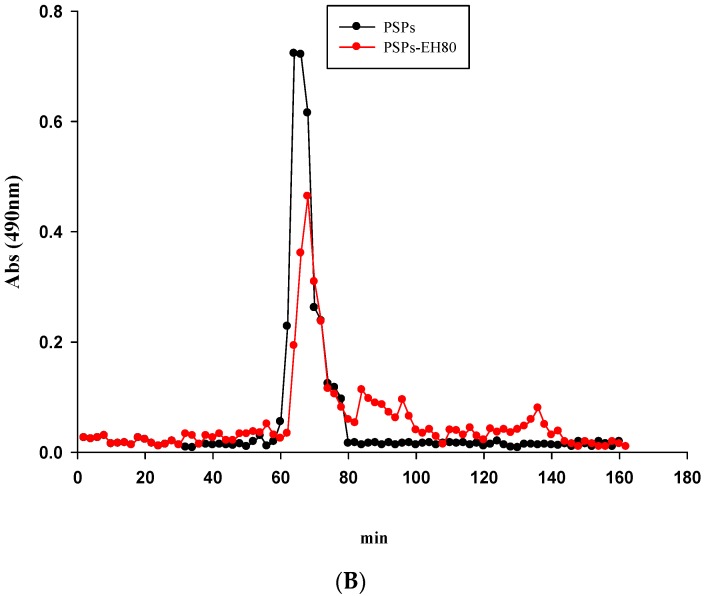
Gel filtration chromatography of the PSPs and PSPs-EH80 on Sepharose CL-6B. The Sepharose CL-6B was calibrated with different molecular masses of dextran (**A**). In this column, the PSPs and PSPs-EH80 elution profiles were determined using the phenol-sulfuric acid method (**B**).

Comparing the antioxidant activity of molecular weight segments between 6.55 to 256 kDa indicated that the 6.55 kDa segment has the strongest antioxidant activity, meaning the polysaccharide antioxidant activity is related to molecular weight [26]. In addition, in the antioxidant activity test, the antioxidant activity of -EH80 of PSP was significantly higher (*p* < 0.05) than PSP-EH20 and PS; then, the CL-6B gel filtration column was used to analyze the molecular weights of PSP and PSP-EH80. Figure 4 shows that the PSPs showed only one peak, which was about 300 kDa, whereas the molecular weight of PSPs-EH80 had four different peaks, at approximately 300, 190, 140 and 50 kDa, respectively. 

The results revealed that β-1,3-glucanase can hydrolyze the macromolecules of PSPs into smaller molecules, and that the hydrolysis rate was 38.6% (Table 1). Our data also showed that the molecular weight of PSPs-EH80 ranged from 50 to 300 kDa. We also suggest that the chelating ability of PSPs-EH80 is related to its low molecular weight.

**Figure 5:**
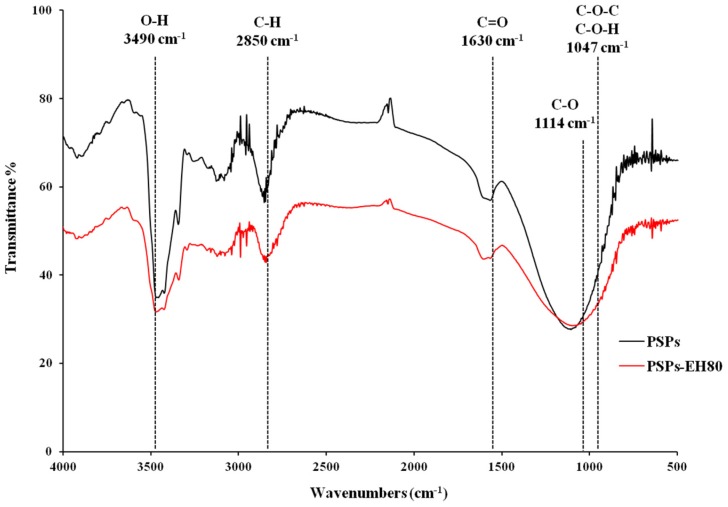
IR spectra of PSPs and PSPs-EH80.FT-IR assignments, wave number (cm^−1^): 3490 stretching vibration (str) of O-H, 2850str of C-H, 1630str of C=O and 1114str of C-O; 1047 is representative of C-O-C and C-O-H.

We identified the organic groups in PSPs and PSP-EH80 using FT-IR spectroscopy. In Figure 5, the band at 3490 cm^−1^ represents the stretching vibration of O-H bonds in constituent sugar residues, while the band at 2850 cm^−1^ is associated with the stretching vibration of C-H bonds in the sugar ring. The O-H and C-H bonds were detected in both the PSPs and PSP-EH80. The characteristic absorption band that appeared at 1630 cm^−1^ was assigned to the stretching of C=O bonds; the band at 1114 cm^−1^ is associated with the stretching vibration of C-O bonds. The C=O and C-O bonds exist in both PSPs and PSPs-EH80. The carbohydrates show a high absorption band between the 1200 cm^−1^ and 850 cm^−1^ regions, referred to as the fingerprint region, where the position and intensity of the bands are specific for every polysaccharide, enabling the identification of major chemical groups in polysaccharides [27,28]. Our results confirmed the polysaccharide pattern in PSPs and PSPs-EH80 (Figure 4).

The C-O-C and C-O-H bonds shown at 1047 cm^−1^, representing a pyran structure, were detected in both the PSPs and PSPs-EH80. According to the spectra in Figure 5, enzymatic digestion did not damage the active site of the sugar chains, and the functional groups of PSPs-EH80 were not changed and remained similar to those of the PSPs.

### 2.5. Effect of PSPs or PSP-EH80 on Cell Viability and ROS Assay in HaCaT Cells

**Figure 6:**
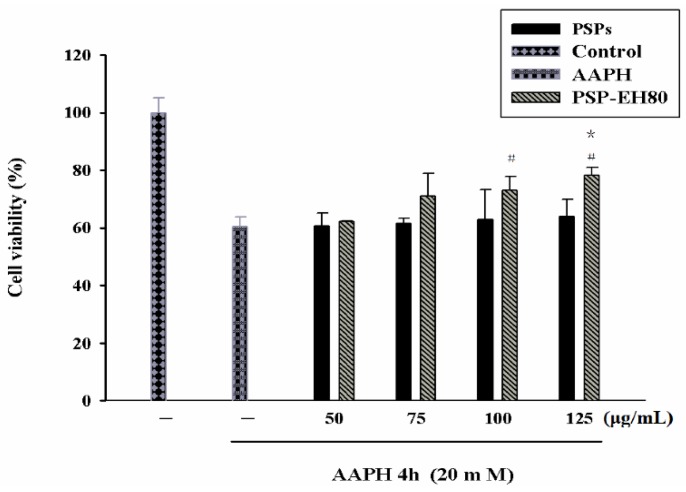
HaCaT cell survival rates after treatment with PSP, PSP-EH80 and AAPH. HaCaT cells (1 × 10^4^) were pre-incubated for 24 h and the cells treated with varying concentrations (μg/mL) of PSPs and PSPs-EH80 for 24 h. AAPH (20 mM) was then added for 4 h at 37 °C in a 5% CO_2_ atmosphere. The absorbance was measured at 570 nm by ELISA. The results are represented as percentages of the control. * *p* < 0.05 compared with the PSPs group; ^#^ *p* < 0.05 compared with the AAPH group.

The cell viability of PSPs or PSP-EH80-treated HaCaT cells was analyzed by a MTT assay. The viability of AAPH-treated cells was 40% lower than that of the control group. PSPs showed little preventive effect on AAPH-induced cell death. PSP-EH80 treatment (75, 100 and 125 μg/mL) increased the viability of AAPH-treated cells in a dose-dependent manner, which was 70%, 73% and 78%, respectively (Figure 6). 

**Figure 7:**
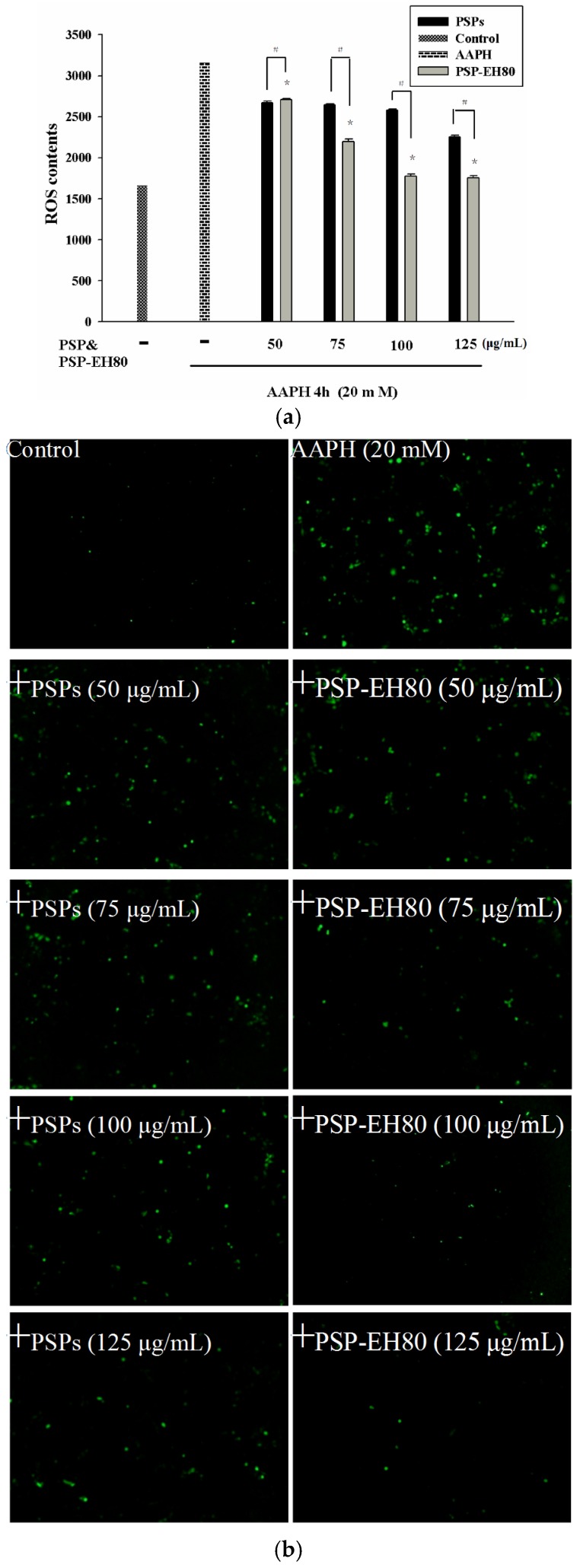
HaCaT cell fluorescence after treatment with PSPs, PSPs-EH80 and AAPH. HaCaT cells (3 × 10^5^) were pre-incubated for 24 h and the cells treated with varying concentrations (μg/mL) of PSP and PSPs-EH80 for 24 h. AAPH (20 mM) was then added for 4 h at 37 °C in a 5% CO_2_ atmosphere. (**a**) The fluorescence intensity of the DCF-stained cells was quantified. * *p* < 0.05 compared with the PSPs group; ^#^ *p* < 0.05 compared with the AAPH group. (**b**) The intracellular ROS level was indicated by DCF fluorescence, and measured using fluorescence microscopy.

Our data suggest that the PSP-EH80 has a better protective effect than PSP on AAPH-induced cell death. Previous studies have reported that AAPH induces oxidative stress through the intracellular elevation of ROS in a variety of cells, including macrophages and keratinocytes [29,30,31]. Increased ROS can break the intracellular antioxidant defense system and cause cell damage and death [32]. In the present study, the oxidative stress induced by AAPH in HaCaT cells was determined by intracellular ROS generation using a DCFH-DA assay. As shown in Figure 7a,b, AAPH increased ROS generation by 2-fold in the HaCaT cells when compared with the control cells. PSP-EH80 treatment (50–125 μg/mL) significantly inhibited AAPH-induced ROS generation in a dose-dependent manner. Our results suggest that PSP-EH80 could prevent cell death induced by AAPH through inhibiting ROS generation. A previous study indicated that polysaccharides with low molecular weight showed better antioxidative ability [11]. Our results also found that the antioxidative effect of PSP-EH80 was better than that of PSPs.

## 3. Materials and Methods

### 3.1. Materials and Components

The fruiting bodies of *Trametes versicolor* (BCRC No.: 35683) were provided by Da Yeh University Biotechnology Research Center; the method of solid-state cultures fruiting bodies was referred to Lekounougou et al. [33]. β-1,3-Glucanase from *Trichoderma longibrachiatum* (CAS Number 9044-93-3), 2,2′-azobis(2-amidinopropane) dihydrochloride (AAPH), 3-(4,5-dimethyl- thiazol-2-yl)-2,5-diphenyltetrazolium bromide (MTT), and 2′,7′-dihydrofluorescein diacetate (DCFH-DA) were purchased from Sigma Chemical Co. (St. Louis, MO, USA). Sepharose CL-6B was purchased from General Electric Healthcare (Chicago, IL, USA). Potassium bromide (IR grade) was purchased from Scharlau Chemie S.A. (Sentmenat, Spain). All of the other chemicals used in this study were of analytical grade and were obtained commercially.

### 3.2. Preparation of PSPs from T. versicolor

The method had been previously used by Huang et al. [2] and Sun et al. [34], with modifications. The fruiting bodies of *T. versicolor* were extracted three times with 80% ethanol under reflux at 75 °C for 6 h to defat them and remove some colored materials, oligosaccharides, lipids and some small molecular materials. The pretreated samples were separated from the organic solvent through a nylon cloth. Each 10 g of dried pretreated sample was extracted by 200 mL water at 95 °C for 2.5 h. The water extraction solutions were separated from the insoluble residue by centrifugation (2000× *g* for 10 min at 20 °C) to remove protein (modified Sevage method) and then precipitated by the addition of dehydrated alcohol to a final concentration of 80% (*v*/*v*). The precipitates collected by centrifugation (2000× *g* for 10 min at 20 °C) were washed with dehydrated alcohol three times and dried under reduced pressure.

### 3.3. Enzymatic Hydrolysis of PSPs

Water extract PSPs were dissolved with 0.1 M sodium acetate buffer solution (pH 5) and made into 2 mg/mL PSPs concentrated solution. Then, 5 mL of β-1,3-glucanase enzyme (20 and 80 units/mL, where one unit was defined as the production of 1 mg of glucose equivalent per min) was added to 5 mL of 2 mg/mL PSPs solution, and the final PSPs concentration was 1 mg/mL. It was mixed well and incubated at 40 °C for 2 h to hydrolyze the PSPs, followed by enzyme inactivation for 5 min at 95 °C to terminate the reaction. The *T. versicolor* PSPs enzymatic hydrolysates 20 U/mL (PSPs-EH20) and 80 U/mL (PSPs-EH80) were then stored at −30 °C [35]. The antioxidant ability of PSPs, PSPs-EH-20 and PSPs-80 was assessed.

### 3.4. Metal Chelating Activity Assay

Each sample (PSPs, PSPs-EH20 and PSPs-EH80), at a different concentration (from 0 to 2.5 mg/mL) (2 mL), was vigorously mixed with 1.7 mL distilled water and 0.1 mL of 2 mM FeCl_2_. The reaction was initiated by adding 0.2 mL of 5mMferrozine, and the mixture was shaken vigorously and left standing at room temperature for 10 min. A control test was performed with distilled water but without a test sample; the solution without ferrozine added was used as a control. The ferrous ion chelating ability was determined by measuring the absorbance at 562 nm using a spectrophotometer (Hitachi U-3010, Tokyo, Japan). The ferrous ion chelating ability was calculated as follows [36]:

Metal chelating activity (%) = [1 − (absorbance of sample − absorbance of blank)/absorbance of control] × 100
(1)

### 3.5. ABTS Radical Scavenging Assay

The ABTS radical cation (ABTS•^+^) solution was prepared by reacting a 7 mM ABTS solution with 2.45 mM potassium persulphate in the dark at room temperature for 12 h. The ABTS•^+^ solution was then diluted with 95% ethanol to obtain an absorbance of 0.7 ± 0.02 at 735 nm. Each sample (PSPs, PSPs-EH20 and PSPs-EH80) was added to 2 mL ABTS•^+^ solution and mixed vigorously. All measurements were taken after at least 6 min. The total antioxidant activity was determined by measuring the absorbance at 735 nm with a spectrophotometer (Hitachi U-3010). A control test was performed with distilled water but without a test sample; the solution, without ABTS added, was used as a control. The ABTS•^+^ radical scavenging activity was calculated as follows [37]:

ABTS radical scavenging activity (%) = [1 − (absorbance of sample − absorbance of blank)/absorbance of control] × 100
(2)

### 3.6. DPPH Radical Scavenging Assay

The DPPH free radical scavenging activity was determined by Yang’s method, with some modifications [38]. Each sample (PSPs, PSPs-EH20 and PSPs-EH80), at a different concentration (from 0 to 2.5 mg/mL) in 2 mL distilled water was mixed with 2 mL 1 mM DPPH solution (dissolved in methanol). The mixture was shaken vigorously and then left to stand in the dark for 30 min. The absorbance of the control sample was accomplished by replacing the test sample with methanol. The DPPH radical scavenging activity was determined by measuring the absorbance at 517 nm using a spectrophotometer (Hitachi U-3010). A control test was performed with distilled water, but without a test sample; the solution without DPPH added was used as a control. The DPPH radical scavenging activity was calculated as follows:

DPPH radical scavenging activity (%) = [1 − (absorbance of sample − absorbance of blank)/absorbance of control] × 100
(3)

### 3.7. Determination of Molecular Weight

The determination of molecular weight followed Lee’s and Zhang’s method, with some modifications [39,40]. The PSPs and PSPs-EH sample fractions were determined by gel filtration with the column packed with Sepharose CL-6B (1.6 × 100 cm), and operated at a flow rate of 0.5 mL/min in distilled water. Standard dextrans T-200, T-150, T-70, T-40 and T-10 were passed through a Sepharose CL-6B column; the elution volumes were then plotted against the logarithms of the respective molecular weights. The elution volumes of PSPs and PSPs-EH samples were plotted on the same graph; the molecular weights of PSPs and PSPs-EH were thereby determined. The hydrolysis rate was calculated using the following formula:

Hydrolysis rate (%) = 1 − [(peak area after hydrolysis/peak area before hydrolysis)] × 100
(4)

### 3.8. FT-IR Spectroscopy

The PSPs and PSPs-EH were individually incorporated into KBr (spectroscopic grade) and pressed into a 1 mm pellet. The spectra were recorded at absorbance ranging from 4000 to 500 cm^−1^ using an FT-IR spectrometer (FT-IR 8400S, Shimadzu, Kyoto, Japan). The FT-IR spectroscopy was determined by Leung’s method, with some modifications [41].

### 3.9. Cell Culture

Human keratinocyte (HaCaT) cell lines were purchased from the American Type Culture Collection (Manassas, VA, USA). HaCaT cells were cultured in Dulbecco’s Modified Eagle’s Medium (DMEM) with 10% fetal bovine serum (FBS) (Life Technologies, Rockville, MD, USA), 100 μg/mL of streptomycin, and 100 units/mL of penicillin (HyClone, Thermo Scientific, Rockford, IL, USA). Cells were maintained in a humidified 5% CO_2_ atmosphere at 37 °C [42].

### 3.10. MTT Assay

Cell viability was determined by using the MTT assay. HaCaT cells at a density of 10^4^ cells/well in 96-well plates were pretreated with various concentrations of PSPs and PSPs-EH80 (50–125 μg/mL) for 24 h, and then incubated with 20 mM AAPH for 4 h. For the MTT assay, 20 μL of MTT solution (5 mg/mL) was added to each well of a 96-well plate and incubated for 2 h. The supernatant was then removed, and the formazan crystals so obtained were dissolved in 200 μL of dimethyl sulfoxide, and quantified by measuring the optical density at 570 nm using an ELISA reader (Thermo). The effect of PSPs on cell viability was assessed as the percent of viable cells compared with the vehicle-treated control cells, which were arbitrarily assigned a viability of 100%. The assay was performed in triplicate at each concentration [29].

### 3.11. Intracellular ROS Detection

The intracellular accumulation of ROS was detected by fluorescence microscopy using DCFH-DA. The HaCaT cells (3 × 10^5^ cells/well) were cultured in a 6-well plate in DMEM supplemented with 10% FBS; the culture medium was renewed when the cells reached 80% confluence. After PSPs and PSPs-EH80 treatment (50–125 μg/mL) for 24 h and AAPH treatment (20 mM) for 4 h, the cells were incubated with 10 μM DCFH-DA in the culture medium at 37 °C for 30 min. The acetate groups on DCFH-DA were then removed by an intracellular esterase, trapping the probe inside the cells. The cells were then washed with warm PBS buffer. ROS production can be measured by the changes in fluorescence due to the intracellular accumulation of 2,7-dichlorofluorescin-diacetate (DCF) caused by the oxidation of DCFH. The DCF fluorescence was measured using a fluorescence microscope (Olympus 1 × 71 at 40× magnification, Tokyo, Japan) [43].

### 3.12. Statistical Analysis

All experiments were carried out in triplicate. All of the data are presented as mean ± standard deviation (SD); differences among the groups were subjected to a one-way ANOVA (analysis of variance) followed by Duncan’s multiple range, Tukey’s HSD or Fisher’s tests (SPSS version 16.0); when statistically significant, they were accepted when a *p*- value < 0.05.

## 4. Conclusions

In this study, the functional groups in PSPs-EH80 did not change during the process of enzymatic hydrolysis with β-1,3-glucanase; in turn, this process could enhance the antioxidant activity of polysaccharides by modification of their molecular weight. PSPs-EH80 also inhibited the oxidative damage induced by ROS on HaCaT cells. In summary, using β-1,3-glucanase in the specific hydrolysis of *T. versicolor* polysaccharides is suitable for enhancing its antioxidative capacity.

## Figures and Tables

**Table 1:** Average molecular weight and hydrolysis rate of PSPs and PSPs-EH80.

PSPs Fraction	Peak	M_w_ (kDa)	Area %	Hydrolysis Rate
PSPs	Peak 1	300 kDa	100.0	38.6%
PSPs-EH80	Peak 2	300 kDa	61.4	
	Peak 3	190 kDa	18.8	
	Peak 4	140 kDa	7.2	
	Peak 5	50 kDa	12.6	
